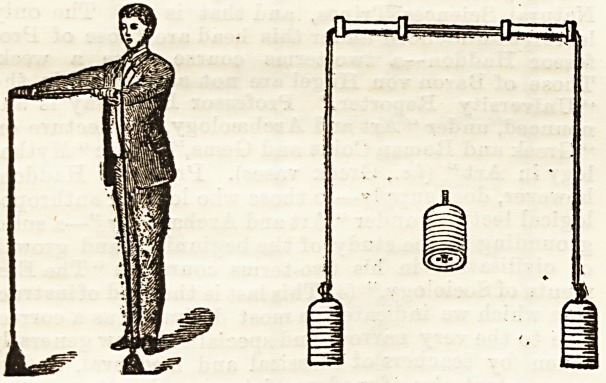# New Appliances and Things Medical

**Published:** 1896-02-01

**Authors:** 


### NEW APPLIANCES AND THINGS MEDICAL.

[We shall be glad to receive, at our Office, 428, Strand, London, W.C., from the manufacturers, specimens of all new preparations and appliances, which may be brought out from time to time.]

### LARGIADÈR'S ARM AND CHEST STRENGTHENER.

(KROHNE AND SESEMANN, 8, Duke Street, Manchester Square, W.)

This new apparatus for home calisthenics, although extensively employed in Germany and foreign orthopædic establishments and gymnasiums, has up to the present obtained little hold on the public in England. The reason is not far to seek; the exercises are founded on scientific principles, which can only be acquired by oral instruction, or from written manuals, and so far all that has been written on the subject has been written in German, and hence for the most part inaccessible to English readers. The apparatus itself consists of two cast-iron weights made up of a number of separate discs, so that the weight can be adjusted to the requirements of the individual. The weights, in their turn, are attached to strong cords, which pass through the centre of one handle to be fixed securely to the other. The range of exercise is almost unlimited, combining, as it does, the varieties peculiar to the dumb-bell, as well as to the ordinary elastic chest-expander used for calisthenic exercises. The applications are multiple, and since any or every group of muscles can be brought into play by the selection of the appropriate exercises, it is equally useful for the treatment of general debility as for special cases of spinal curvature or other deformity. During the exercises the respiratory movements are so ordered as to further the ventilation of the lungs, and ensure that free expansion of the apices which is probably the safest prophylactic measure against tubercular deposits in this situation known to medical science. Messrs. Krohne and Sesemann, who are the London agents for this useful invention, are preparing descriptive literature and full instructions for use. Those who can read German could do no better than read Zahn's handbook on the subject. The only criticism we have to make is on the practical working of this Largiadèrs apparatus. The ropes do not run through the handles with sufficient ease; the undue friction might easily be obviated by the fixation of a couple of small pulleys.

### COCA-TONIC WINE ("OTHNIEL" BRAND).

(H. E. STEVENSON AND CO., 130, SOUTHWARK STREET, LONDON, S.E.)

There is little need at the present time to expatiate on the virtues of the alkaloids which exist in the leaves of erythroxylon coca. The various coca wines are a result of the recognition of this fact, and we are glad to be able to add our testimony to the excellency of yet another brand of coca wine which has recently been put upon the market by Messrs. H. E. Stevenson and Co., of Southwark Street. The Othniel Coca Wine has a dark brown sherry colour and possesses a very marked flavour of the tannin and principles of the coca leaves. Our analysis shows that the process adopted by the manufacturers has resulted in a very full extraction of the active principles of the leaves, as our analyst finds that the total extract amounts to no less than 12·26 per cent. of the wine. That this is mainly the tannin, alkaloids, and other organic constituents of the coca leaves is shown further by the fact that the mineral constituents of this extract are low, amounting to only 0·29 per cent. calculated on the original sample of wine. A determination of the alkaloids present show that at least 3 grams weight of average coca leaves must have been used in preparing every 100 cubic centimetres of the wine. The alcoholic strength was found to be 16·54 per cent. absolute alcohol by weight, but the acidity of the wine was high, if regarded in the light of a dessert wine, but probably of no material significance in a medicinal preparation. The total acidity was equivalent to 80 c.c. of decinormal alkali per 100 c.c., and of this amount about one-third was acetic acid, or other volatile acid, as the distillate from 100 c.c. of the wine required 30·4 c.c. of standard alkali for neutralisation. We can quite believe that a wineglassful acts as a prompt and powerful restorative in bodily and mental fatigue, nervous exhaustion, sleeplessness, and the effects of overwork and worry.

## Figures and Tables

**Figure f1:**